# Giant Renal Cyst Causing Malnutrition and Weight Loss: A Case Report

**DOI:** 10.1155/crgm/6635819

**Published:** 2025-09-09

**Authors:** Kemal Ertaş, Abdullah Akkurt

**Affiliations:** ^1^Department of Urology, Şırnak State Hospital, Şırnak, Turkey; ^2^Department of Urology, Dr. Gazi Yaşargil Training and Research Hospital, Diyarbakır, Turkey

**Keywords:** giant renal cyst, laparoscopy, malnutrition, weight loss

## Abstract

Simple renal cysts are the most common cystic abnormalities of the kidney, typically observed in older individuals and often asymptomatic, requiring no treatment. These cysts are usually detected incidentally during imaging for unrelated conditions. Giant renal cysts, defined as those exceeding 15 cm in diameter and containing over 1500 mL of serous fluid, are exceptionally rare. We report a case of a 34-year-old male presenting with a rapidly growing giant renal cyst (40 × 28 cm) in the left kidney, resulting in malnutrition and significant weight loss. The patient underwent successful laparoscopic transperitoneal cyst excision. At the 1-year follow-up, the patient was asymptomatic, with no evidence of residual cyst recurrence.

## 1. Introduction

Simple renal cysts are the most prevalent type of acquired renal cysts, typically identified incidentally during radiologic imaging for extrarenal conditions. These cysts are often asymptomatic, with the prevalence increasing with age and a tendency for more rapid growth in younger patients [1, 2]. They are predominantly located in the renal cortex, and their etiology remains incompletely understood, though they are hypothesized to originate from diverticula of the distal convoluted tubule [3]. Emerging evidence suggests an association between simple renal cysts and hypertension [4]. Giant renal cysts, measuring over 15 cm in diameter and containing more than 1500 mL of serous fluid, are exceedingly rare [5].

While most simple renal cysts are asymptomatic, large cysts may cause symptoms such as dull back or flank pain, fever (in cases of infection or malignancy), or upper abdominal pain [1]. Symptomatic cysts or those associated with complications, such as hypertension or hydronephrosis, may necessitate intervention [6]. Laparoscopic management of symptomatic giant renal cysts is a safe and effective approach, with low rates of recurrence and morbidity [7].

## 2. Case Report

A 34-year-old male presented to the outpatient clinic with a 3-month history of progressive abdominal pain, fatigue, abdominal distension, malnutrition, and unintentional weight loss of approximately 10 kg. The patient reported inadequate nutritional intake during this period. On physical examination, the patient's height was 182 cm, weight was 60 kg, and body mass index (BMI) was 18.1 kg/m^2^. There was marked asymmetrical distension on the left side of the abdomen. The abdominal wall was tense but nontender on palpation and percussion. Laboratory investigations, including routine hematology, biochemistry, and serum tumor markers (AFP, TPSA, β-HCG, CEA, CA19-9, and CA-15-3), were within normal limits. Serum albumin was 3.2 g/dL, and prealbumin was 13 mg/dL, indicating malnutrition.

**Figure 1:**
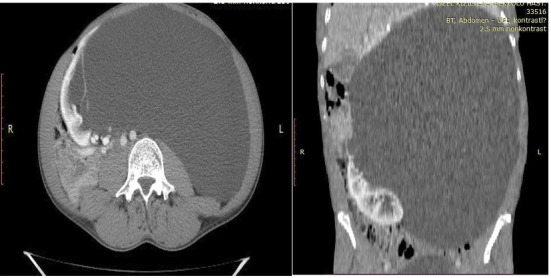
Computed tomography scan of our patient. Sagittal and coronal views of computed tomography of the abdomen and pelvis demonstrate a large cyst arising from the interpolar region of the left kidney. The cyst measures 40 × 28 cm in craniocaudal and transverse dimensions, respectively.

Abdominal computed tomography (CT) revealed a giant Bosniak Type II renal cyst measuring 40 × 28 cm, occupying the abdominal cavity and displacing intra-abdominal organs to the right, mimicking ascites (Figure 1). Differential diagnosis included hydatid cyst and malignancy. An Echinococcus agglutination test was negative, ruling out hydatid disease. Fluid aspiration from the cyst was performed, and cytological analysis showed no malignant cells. Analysis of the cyst fluid for urea and creatinine excluded urinoma, confirming the diagnosis of a simple renal cyst.

**Figure 2:**
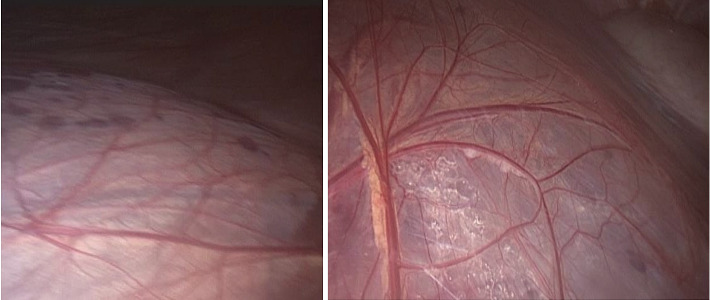
General view of the cyst.

**Figure 3:**
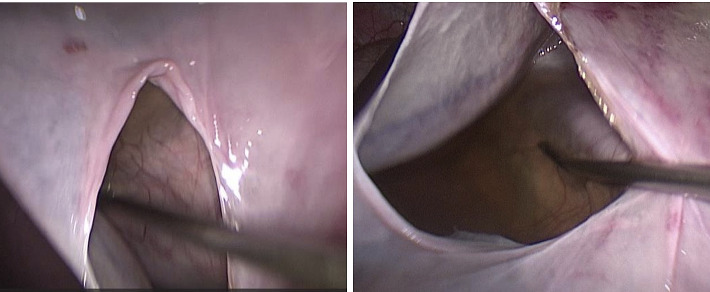
View of the roof of the emptied cyst.

**Figure 4:**
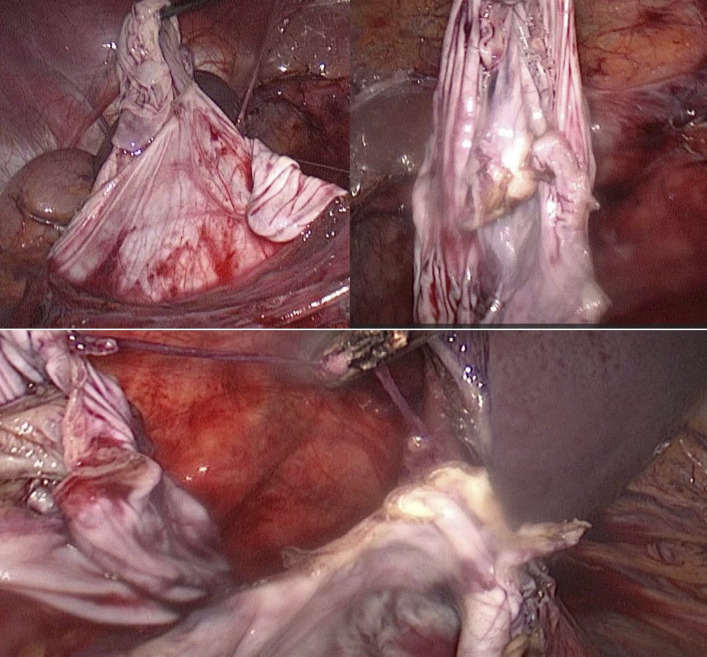
Excision of the renal cyst wall.

Due to significant abdominal distension, safe laparoscopic entry was challenging. Under general anesthesia, the patient was positioned laterally with the cyst side elevated. An 18-gauge needle was inserted posteriorly to aspirate approximately 2000 mL of fluid, facilitating safe abdominal access. Pneumoperitoneum was established using a Veress needle. Three ports were placed: a 10-mm port for a 30- laparoscope near the lateral border of the rectus abdominis muscle, a 5-mm midline port 10 cm above the umbilicus, and a 5-mm port in the midaxillary line. The giant cyst was identified, displacing abdominal organs (Figure 2). Direct dissection was performed without medializing the colon, using a blunt grasper and ligature. After evacuating the cyst contents (approximately 9000 mL of serous fluid), the cyst roof was excised 5 mm from the renal parenchyma using a ligature (Figures 3 and 4). The specimen was sent for histopathological examination. Hemostasis was achieved with electrocoagulation of the cyst margins, avoiding the base to prevent renal parenchymal damage. A 16-Fr polyethylene drain was placed via the lateral 5-mm trocar, and the procedure was completed with desufflation and cosmetic closure of the ports.

**Figure 5:**
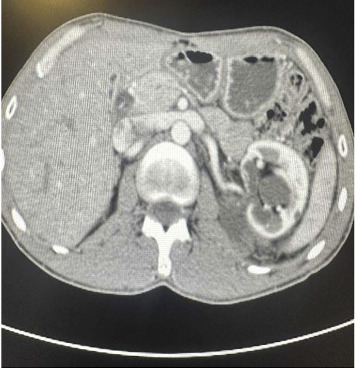
Subsequent computed tomography after one year revealing quasi-normal aspect of the kidneys.

The transperitoneal laparoscopic cyst excision was completed successfully in approximately 55 min. The patient recovered uneventfully and was discharged after one night. Histopathology confirmed a simple multilocular renal cyst. At the 6-month follow-up, the patient had regained approximately 10 kg, with stable weight thereafter. At the 1-year follow-up, the patient remained asymptomatic, with no evidence of cyst recurrence (Figure 5).

## 3. Discussion

Simple renal cysts are among the most common benign renal lesions, typically smaller than 2 cm and often impalpable [1, 5]. Their etiology remains unclear, with developmental cysts potentially arising from persistent urinary tubule abnormalities and acquired cysts linked to focal ischemia or inflammation [8, 9]. Giant renal cysts exceeding 15 cm are exceptionally rare [10]. Large cysts may cause symptoms by compressing adjacent structures, leading to abdominal distension, pain, early satiety, constipation, hypertension, or, in rare cases, infection-related complications such as pyelonephritis [11].

In this case, the 40 × 28-cm cyst caused significant compression of the stomach, resulting in early satiety, malnutrition, and a 10-kg weight loss over 3 months. The cyst's size and radiologic appearance initially mimicked ascites [12], raising concerns for peritonitis carcinomatosis or gastrointestinal malignancy. This presentation underscores the importance of including giant renal cysts in the differential diagnosis of abdominal distension.

Treatment options for symptomatic renal cysts include percutaneous aspiration with or without sclerotherapy, percutaneous marsupialization, open surgery, or laparoscopic surgery via transperitoneal or retroperitoneal approaches [1, 13]. Percutaneous aspiration with sclerotherapy (e.g., alcohol injection) is effective for smaller cysts but may be inadequate for giant cysts with residual volumes exceeding 200 mL, necessitating surgical intervention [14]. Laparoscopic cyst excision is a well-established, minimally invasive approach for giant cysts, offering low recurrence and morbidity rates [6, 15, 16]. In this case, transperitoneal laparoscopic excision was performed successfully, marking it as the largest reported renal cyst managed laparoscopically.

To facilitate safe trocar placement in the presence of significant abdominal distension, preoperative cyst aspiration of 2000 mL was performed, a technique not commonly reported but effective in this context. This approach highlights the feasibility of laparoscopic management for giant renal cysts, even in challenging cases, provided there is sufficient expertise.

## 4. Conclusion

Giant renal cysts are a rare cause of significant clinical symptoms, including malnutrition and weight loss due to gastric compression, as observed in this case. To our knowledge, this 40 × 28-cm cyst is the largest reported renal cyst managed laparoscopically. Transperitoneal laparoscopic cyst excision is a safe and effective treatment option for giant renal cysts, with minimal morbidity and low recurrence rates. Further studies are needed to elucidate the etiology of rapid cyst growth, particularly in younger patients.

## Data Availability

The data that support the findings of this study are available on request from the corresponding author. The data are not publicly available due to privacy or ethical restrictions.
